# Analysis of variation in bronchovascular pattern of the right middle and lower lobes of the lung using three-dimensional CT angiography and bronchography

**DOI:** 10.1007/s11748-017-0754-4

**Published:** 2017-02-14

**Authors:** Toshiteru Nagashima, Kimihiro Shimizu, Yoichi Ohtaki, Kai Obayashi, Seshiru Nakazawa, Akira Mogi, Hiroyuki Kuwano

**Affiliations:** Division of General Thoracic Surgery, Integrative Center of General Surgery, Gunma University Hospital, 3-39-22 Showa-machi, Maebashi, Gunma 371-8511 Japan

**Keywords:** Lung anatomy, Three-dimensional CT, Lung cancer surgery, Segmentectomy

## Abstract

**Objectives:**

General thoracic surgeons must be familiar with anatomical variation in the pulmonary vessels and bronchi. Here, we analyzed the bronchovascular pattern of the right middle lobe (RML) and right lower lobe (RLL) of the lung using three-dimensional CT angiography and bronchography (3DCTAB).

**Methods:**

We reviewed the anatomical patterns of the pulmonary vessels and bronchi in 270 patients using 3DCTAB images.

**Results:**

The branching patterns of vessels and bronchi of RML and S^6^ were classified according to the number of stems. The single-stem type was the most common, except in the artery of the RML, for which the two-stem type was the most common. The artery and bronchus of S*, which is an independent segment between S^6^ and S^10^, were observed in 20.4% of cases. The branching pattern of A^7^ (B^7^) was classified into four types. The A^7^a (B^7^a) type was observed in 74.8% of cases, and was the most common. The branching pattern of the artery and bronchus of S^8−10^ was classified into five and three types, respectively. The A^8^ and A^9^ + A^10^ type, and the B^8^ and B^9^ + B^10^ type, were observed in 68.1 and 80.4% of cases, respectively, and were the most common types. The branching pattern of V^8−10^ was more complex than that of A^8−10^ and B^8−10^.

**Conclusion:**

We explored the bronchovascular patterns of RML and RLL and their frequencies using a large number of 3DCTAB images. Our data can be used by thoracic surgeons to perform safe and precise lung resections.

**Electronic supplementary material:**

The online version of this article (doi:10.1007/s11748-017-0754-4) contains supplementary material, which is available to authorized users.

## Introduction

For thoracic surgeons, knowledge of pulmonary bronchovascular patterns, including rare anatomical variations, is extremely important to perform safe and accurate anatomical pulmonary resections. However, currently available anatomical data on pulmonary bronchovascular patterns are limited to a few cadaveric studies. These studies include the small number of cases reported by Boyden et al. [1, 2] and Yamashita [3], performed between the 1950s and 1970s. In a previous study, we focused on the usefulness of 3DCT imaging to understand individual variation in thoracic anatomy, and previously reported a systematic radiological analysis of the pulmonary structure of the right upper lobe (RUL) using the largest number of cases in the literature (*n* = 263) [4]. In this study, we analyzed variation in the pulmonary bronchovascular pattern using 3DCT angiography and bronchography (3DCTAB) of the right middle lobe (RML) and right lower lobe (RLL) of the lung.

## Patients and methods

### Reconstruction of 3DCTAB imaging

Bronchovascular patterns revealed by 3DCTAB imaging were analyzed by 64-channel multidetector row computed tomography (MDCT) (SOMATOM Definition Flash; Siemens Healthcare, Berlin, Germany). A total of 35 mL contrast agent was mechanically injected at 5 mL/s, immediately followed by injection of 20 mL of saline. A solid image was constructed from 1.0-mm data slices of contrast-enhanced CT images with the aid of 3D volume rendering. The volume data from both arterial and venous phases were transferred to a workstation running volume-rendering reconstruction software (Ziostation2; Ziosoft, Tokyo, Japan) that converted the data into the 3DCT angiographic format. The 3D reconstruction of the bronchial tree involved mathematical morphology-based 2D segmentation of axial images, followed by restoration via manual addition of segments from 2D axial images to form 3D images. Radiology technicians processed all 3D images, and thoracic surgeons confirmed the validity of the reconstructed images. The detection rate of pulmonary vessels by our 3DCTAB was 98.7% [4], indicating the feasibility of comparing our 3DCTAB data to previously reported data based on actual anatomy.

### Patient preparation and examination

Between November 2010 and November 2015, 302 patients with respiratory or mediastinal lesions underwent 3DCTAB prior to surgery. Thirty-two cases were excluded because some subsegmental branches of the pulmonary vessels and bronchi were not adequately represented on 3DCTAB due to technical issues, such as obstruction by lung tumors and/or lymph node metastasis. Therefore, we analyzed variation in the pulmonary vessel patterns of the most recent 270 consecutive cases (150 men, 120 women; median age 67 years) in whom all subsegmental branches were properly represented on 3DCTAB. Patient characteristics are summarized in Supplemental Table 1.

The frequencies of each bronchovascular pattern in our study and those of previous studies (Boyden et al. [1, 2] and Yamashita [3]) were compared using the *χ* ^2^ test. However, some branching patterns were not described in these previous reports [1–3], and thus our data could not compare for those patterns. All statistical analyses were performed using SPSS ver. 22 software (SPSS, Inc., Chicago, IL, USA). This study was approved by the Research Ethics Committee at Gunma University Hospital, Maebashi, Gunma, Japan.

### Definition of pulmonary vessels and bronchi

We used the same nomenclature to describe segmental structures used by Boyden et al. [1, 2] and Yamashita [3]. Segmental and subsegmental vessels were named with reference to their relationships to the segmental bronchi.

We classified the branching patterns of vessels and bronchi in the RML and S^6^ according to the number of stems. However, the bronchovascular pattern of the right basal segments (S7, S8, S9, and S10) was more complex than those of the RML and S^6^. Therefore, we defined the pattern of the right basal segment according to Yamashita’s [3] classification as follows:

Subsuperior segment (S*) The independent segment that is infrequently observed between S^6^ and S^10^ is called the subsuperior segment, S* (Suppl Fig. 1). S* has an independent segmental bronchus (B*) and artery (A*) that are distinct from those of S^6^ and S^10^. B* (A*) bifurcates from between the basal bronchus (artery) and B^10^ (A^10^), and B* (A*) points in a posterolateral direction directly toward the vertebral body.

*Mediobasal segment (S* ^*7*^ *)* A^7^ (B^7^) divides into an anterior ramus A^7^a (B^7^a) and a posterior ramus A^7^b (B^7^b). A^7^a (B^7^a) runs anterior to the inferior pulmonary vein (IPV), whereas A^7^b (B^7^b) runs posterior to the IPV. A^7^ (B^7^) is classified into four types according to the combination of A^7^a (B^7^a) and A^7^b (B^7^b). The A^7^a (B^7^a) type has only A^7^a (B^7^a), and in this type, S^7^ is located anterior to IPV. The A^7^b (B^7^b) type has only A^7^b (B^7^b), and in this type, S^7^ is located posterior to IPV. The A^7^ab (B^7^ab) type has both A^7^a (B^7^a) and A^7^b (B^7^b), and in this type, S^7^ is located both anterior and posterior to IPV. Lack of an original A^7^ (B^7^) was occasionally observed. In such instances, S^7^ was supplied by an accessory artery (bronchus) branching from A^8^ (B^8^), A^9^ (B^9^), or A^10^ (B^10^). This type was named the AX^7^ (BX^7^) type. B^7^ was classified in accordance with the artery.

*Ventrobasal, laterobasal, and dorsobasal segments (S* ^*8*^, *S* ^*9*^, *S* ^*10*^ *)* The branching patterns of the ventrobasal, laterobasal, and dorsobasal segmental arteries and veins were classified into two types: bifurcation and trifurcation types. The bifurcation type was further divided into two subtypes: simple and split. In the simple bifurcation type, each basal segment was supplied by a single segmental artery: A^8^ and A^9^ + A^10^ type and A^8^ + A^9^ and A^10^ type. In the split type, S^8^ or S^9^ was supplied by two segmental arteries: A^8^ and A^8^ + A^9^ + A^10^ type and A^8^ + A^9^ and A^9^ + A^10^ type. The ventrobasal, laterobasal, and dorsobasal segmental veins were also classified according to the artery. In the simple type, each basal segment was drained by a single segmental vein: V^8^ and V^9^ + V^10^ type and V^8^ + V^9^ and V^10^ type. In the split type, S^9^ or S^10^ was drained by two segmental veins: V^8^ + V^9^ and V^9^ + V^10^ type and V^8^ + V^9^ + V^10^ and V^10^ type. The ventrobasal, laterobasal, and dorsobasal segmental bronchi were also classified in accordance with vessels. However, there were no split bifurcation types.

## Results

### Right middle lobe

*Artery (A* ^*4*^ *and A* ^*5*^ *)* The branching pattern of the RML artery was divided into three types according to the number of stems (Suppl Fig. 2; Suppl Table 2). In 188 of 270 cases (69.6%), the RML artery had two stems, which was the most common type (Suppl Fig. 2b). This type was seen significantly more frequently than noted in previous studies (Yamashita 53.4%; *p* = 0.002, Boyden [1] 52%; *p* = 0.015). The single-stem type and three-stem type were observed in 79 cases (29.3%) and 3 cases (1.1%), respectively (Suppl Fig. 2a, c), and were seen significantly less frequently than in previous studies (Yamashita 42.5%; *p* = 0.01, Boyden [1] 48%; *p* = 0.009, and Yamashita 4.1%; 0.049, Boyden [1] 0%). A new uncommon A^4^ branching pattern was identified in one case (0.4%); A^4^ branched from A^7^, and this pattern was not noted in any of three previous reports (Suppl Fig. 3).

**Fig. 1:**
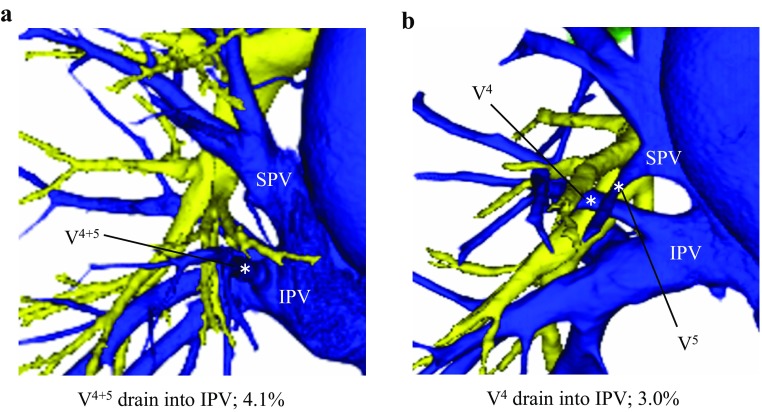
Uncommon drainage patterns of veins in the right middle lobe. **a** Aberrant V^4+5^. **b** Aberrant V^4^. *SPV* superior pulmonary vein. *IPV* inferior pulmonary vein

*Vein (V* ^*4*^ *and V* ^*5*^ *)* The branching pattern of the RML vein was divided into three types according to the number of stems (Suppl Fig. 2; Suppl Table 2). In 177 of 270 cases (67.7%), the RML vein had a single stem, which was the most common type (Suppl Fig. 2d). This type was seen significantly more frequently than noted by Yamashita (45.6%; *p* = 0.002). The two-stem type was seen in 87 cases (30.7%) (Suppl Fig. 2e). The three-stem type was observed only in six cases (1.6%) (Suppl Fig. 2f), and was seen significantly less frequently than in previous studies (Yamashita 8.8%; *p* = 0.005, Boyden [1] 12%; 0.001). Some uncommon drainage patterns of the RML vein were also identified. In 19 cases (7.1%), V^4^ plus V^5^ or part of the middle lobe vein (V^4^ or V^5^) drained into the IPV, and we named these uncommon veins as aberrant V^4+5^ and aberrant V^4^ or V^5^ (Fig. 1a, b); aberrant V^4+5^ was found in 11 cases (4.1%) and aberrant V^4^ or V^5^ was found in 8 cases (3.0%).

*Bronchus (B* ^*4*^ *and B* ^*5*^ *)* In B^4+5^, all cases had a single stem without any variation (Suppl Table 2).

### Right lower lobe

#### The pulmonary artery

**Fig. 2:**
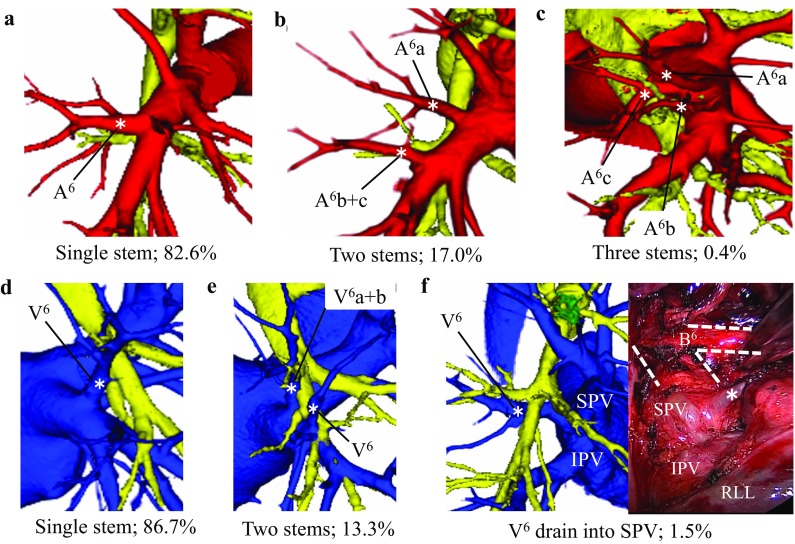
Branching patterns of the superior segmental artery (A^6^) in the right lower lobe. **a** Single stem. **b** Two stems. **c** Three stems. Branching patterns of the superior segmental vein (V^6^) in the right lower lobe. **d** Single stem. **e** Two stems. **f** V6 draining into SPV, i.e., aberrant V^6^. *RLL* right lower lobe

*Superior segmental artery (A* ^*6*^ *)* The branching pattern of A^6^ was divided into three types according to the number of stems (Fig. 2a–c; Suppl Table 3). In 223 cases (82.6%), A^6^ had a single stem, which was the most common type (Fig. 2a). The two-stem type was observed in 46 cases (17.0%) (Fig. 2b), and the three-stem type was observed in only 1 case (0.4%) (Fig. 2c). Our data did not differ significantly from those of Yamashita.

Subsuperior segmental artery (A*) A* was detected in 55 cases (20.4%) (Suppl Fig. 4; Suppl Table 3), which was almost the same as the rate reported by Yamashita, but lower than that reported by Boyden (48%; *p* < 0.001) [2]. In 52 of 55 cases, A* had a single stem, which was the most common type (Suppl Fig. 4a). In 3 of 55 cases, A* had two stems, and this type was seen significantly less frequently than reported by Yamashita (4.6%; *p* = 0.027) (Suppl Fig. 4b).

**Fig. 3:**
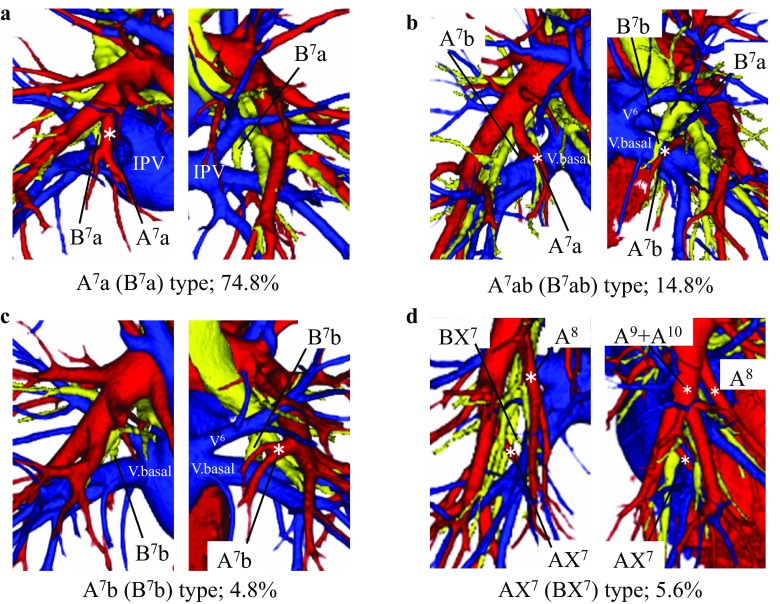
Branching patterns of the mediobasal segmental artery (A^7^) and bronchi (B^7^) in the right lower lobe. **a** A^7^a (B^7^a) type. **b** A^7^ab (B^7^ab) type. **c** A^7^b (B^7^b) type. **d** AX^7^ (BX^7^) type. *V. basal* basal vein

*Mediobasal segmental artery (A* ^*7*^ *)* The branching pattern of A^7^ was divided into four types (Fig. 3; Suppl Table 3). The A^7^a type was observed in 202 cases (74.8%), which was the most common type (Fig. 3a). This type was significantly more frequent than in the previous reports (Yamashita 53.7%; *p* < 0.001, Boyden [2] 56%; *p* = 0.007). On the other hand, the A^7^ab type was observed in 40 cases (14.8%) (Fig. 3b), and its frequency was significantly lower than that reported by Yamashita (28.5%, *p* = 0.001). The A^7^b type was evident in 13 cases (4.8%) (Fig. 3c), and the AX^7^ type was evident in 15 cases (5.6%) (Fig. 3d). The most common supplying artery for AX^7^ type was A^10^, which was seen in 10 cases. Among the remaining five cases, we identified one case supplied from A^8^, two from A^9^, and two from both A^8^ and A^10^. According to Yamashita’s classification, A^7^b was defined as the artery that crosses over and runs posterior to IPV. However, in 46 (37 cases of A^7^ab type, 9 cases of A^7^b type) of the 53 A^7^b cases (87%), A^7^b ran not over IPV but between V^6^ and the basal pulmonary vein (Fig. 3b, c). In fact, A^7^b running over IPV as described by Yamashita was seen in only 7 of 53 A^7^b cases (13%) in the present study.

*Ventrobasal, laterobasal, and dorsobasal segmental arteries (A* ^*8*^, *A* ^*9*^, *A* ^*10*^ *)* The bifurcation type was observed in 259 cases (95.9%) (Suppl Fig. 5a–d; Suppl Table 3). The simple type was observed in 214 cases (79.2%), and was further divided into two subtypes. The A^8^ and A^9^ + A^10^ type was observed in 184 cases (68.1%) (Suppl Fig. 5a), which was the most common type. However, its frequency was significantly lower than that reported by Yamashita (90.8%; *p* < 0.001). The A^8^ + A^9^ and A^10^ type was observed in 30 cases (11.1%) (Suppl Fig. 5b). The split bifurcation type was observed in 45 cases (16.7%), and this variation was not reported previously. This variation was also further divided into two subtypes. The A^8^ and A^8^ + A^9^ + A^10^ type was observed in 38 cases (14.1%) (Suppl Fig. 5c), and the A^8^ + A^9^ and A^9^ + A^10^ type was observed in 7 cases (2.6%) (Suppl Fig. 5d). The trifurcation type (A^8^ and A^9^ and A^10^) was observed in only 11 cases (4.1%) (Suppl Fig. 5e).

#### The pulmonary vein

*Superior segmental vein (V* ^*6*^ *)* The branching pattern of V^6^ was divided into two types (Fig. 2d–e; Suppl Table 4) according to the number of stems. In 234 cases (86.7%), V^6^ had a single stem, which was the most common type (Fig. 2d). V^6^ with two stems was observed in 36 cases (13.3%) (Fig. 2e). Our data did not differ significantly from the previous reports. An uncommon drainage pattern of V^6^ was also identified, in which V^6^ drained into the superior pulmonary vein (SPV), running behind the intermediate bronchus. This variation was not reported previously. We named this newly identified uncommon vein, which was seen in four cases (1.5%), aberrant V^6^ (Fig. 2f).

Subsuperior segmental vein (V*) The V* frequently drained into V^6^ or V^10^. Furthermore, it did not have an independent intersegmental vein, and the pattern of V* was very complex. Therefore, we could not classify its branching pattern.

*Mediobasal segmental vein (V* ^*7*^ *)* Most branches of V^7^ were thin and drained into various parts of the IPV, so we could not strictly classify its branching pattern. V^7^ usually has two branches: V^7^a, which is an intersubsegmental vein between S^7^a and S^7^b; V^7^b, which is an intersegmental vein between S^7^ and S^10^.

*Ventrobasal, laterobasal, and dorsobasal segmental veins (V* ^*8*^, *V* ^*9*^, *V* ^*10*^ *)* The bifurcation type was observed in 263 cases (97.4%) (Suppl Fig. 6a–e; Suppl Table 4). The simple bifurcation type was observed in 123 cases (45.6%), and was further divided into two subtypes. The V^8^ + V^9^ and V^10^ type was observed in 68 cases (25.2%) (Suppl Fig. 6a), and the V^8^ and V^9^ + V^10^ type was observed in 55 cases (20.4%) (Suppl Fig. 6b). The split type was observed in 140 cases (51.8%), and this variation was also further divided into two subtypes. The V^8^ + V^9^ + V^10^ and V^10^ type was observed in 84 cases (31.1%) (Suppl Fig. 6c), and was identified significantly more frequently than in the previous reports (Yamashita 14.2%; *p* < 0.001, Boyden [2] 14%; *p* = 0.014). On the other hand, the V^8^ + V^9^ and V^9^ + V^10^ type was observed in 56 cases (20.7%) (Suppl Fig. 6d), and the frequency of this type was significantly lower than those in the previous reports (Yamashita 33.8%; *p* = 0.005, Boyden [2] 44%; *p* < 0.001). We did not encounter the V^8^ + V^9^ and V^9^ + V^10^ type (as observed in 6.5% of the cases reported by Yamashita and in 4% of the cases reported by Boyden [2]). The trifurcation type (V^8^ and V^9^ and V^10^) was observed in only seven cases (2.6%) (Suppl Fig. 6e).

#### Bronchus

*Superior segmental bronchus (B* ^*6*^ *)* The branching pattern of B^6^ was divided into two types (Suppl Fig. 7a, b; Suppl Table 5) according to the number of stems. In 264 cases (97.8%), B^6^ had a single stem (Suppl Fig. 7a). In only six cases (2.2%), B^6^ had two stems, and the frequency of this type was significantly lower than that reported by Boyden [2] (8%, *p* = 0.031) (Suppl Fig. 7b). The two-stem type was not reported by Yamashita.

Subsuperior segmental bronchus (B*) B* was detected in 55 cases (20.4%), and was similar to A* (Suppl Fig. 4; Suppl Table 5). However, in all of the B* cases, B* had only a single stem unlike A*. The frequency of B* cases did not differ from that reported by Yamashita, but was significantly lower than that reported by Boyden [2] (48%; *p* < 0.001).

*Mediobasal segmental bronchus (B* ^*7*^ *)* The branching pattern of B^7^ was identical to that of A^7^ and was divided into four types (Fig. 3; Suppl Table 5). The B^7^a type was seen in 202 cases (74.8%) (Fig. 3a), and was the most common type. This type was significantly more frequent than in the previous reports (Yamashita 53.7%; *p* < 0.001, Boyden [2] 22%; *p* < 0.001). The B^7^ab type was observed in 40 cases (14.8%) (Fig. 3b), and was significantly less frequent than in the previous reports (Yamashita 28.5%; *p* = 0.001, Boyden [2] 58%; *p* < 0.001). In all cases of the B^7^ab type, B^7^a and B^7^b branched as a common trunk unlike the artery. The B^7^b type was observed in 13 cases (4.8%) (Fig. 3c), and the BX^7^ type was observed in 15 cases (5.6%) (Fig. 3d). According to Yamashita’s classification, B^7^b was defined as the bronchus that crosses over and runs posterior to IPV. However, in all B^7^b cases, B^7^b did not cross over to the IPV but rather ran between V^6^ and the basal pulmonary vein (Fig. 3b, c).

*Ventrobasal, laterobasal, and dorsobasal segmental bronchi (B* ^*8*^, *B* ^*9*^, *B* ^*10*^ *)* The branching pattern of the right basal bronchus was classified into three types (Suppl Fig. 7c–e; Suppl Table 5). The B^8^ and B^9^ + B^10^ type was observed in 217 cases (80.4%) (Suppl Fig. 7c), and was the most common type. However, this type was seen significantly less frequently than in the previous reports (Yamashita 90.8%; *p* = 0.008, Boyden [2] 94%; *p* = 0.020). The B^8^ + B^9^ and B^10^ type was observed in 41 cases (15.2%) (Suppl Fig. 7d), and was significantly more frequent than in Yamashita’s report (6.9%; *p* = 0.019). The B^8^ and B^9^ and B^10^ type was observed in 12 cases (4.4%) (Suppl Fig. 7e).

## Discussion

We performed systematic radiological analyses of the pulmonary structure of the RML and RLL, and examined the discrepancies between our results and those of previous reports [1–3]. Only a few reports have focused on thoracic anatomical abnormalities detected with the aid of 3DCT [5, 6]. However, there have been no previous systematic explorations of the bronchovascular pattern using 3DCT imagery. Therefore, this is the first systematic radiological analysis of the pulmonary structure of the RML and RLL. Moreover, our study included a larger number of cases (*n* = 270) than all previous relevant reports.

The frequencies of some branching types differed from those described in previous reports [1–3], and these discrepancies were particularly evident for the basal segment. Moreover, the bronchovascular pattern of S^7^ was more complex than that of S^8−10^ because the branching pattern of B^7^ (and A^7^) depends on its relationship with IPV and/or the basal vein. In our data, 74.8% of all cases were of the B^7^a type, and the frequency of this type was significantly higher than in previous reports (Yamashita 53.7%; *p* < 0.001, Boyden [2] 22%; *p* < 0.001). On the other hand, the B^7^ab type and the B^7^b type were observed at rates of 14.8 and 4.8%, respectively. The frequencies of these types were significantly lower than those reported by Yamashita (28.5%; *p* = 0.001, 10.0%; *p* = 0.049). Our results for B^7^ differ from those of Yamashita mainly because they defined B^7^b and B* as equal, whereas we clearly distinguished between B^7^b and B*. Both B* and B^7^b bifurcate from the basal bronchus from similar sites; however, B* points in the posterolateral direction directly toward the vertebral body, whereas B^7^b points in the mediobasal direction. Furthermore, B^7^b mainly crossed over the basal pulmonary vein in our study, unlike in previous reports. The advantage of our study using 3DCTAB was that we could analyze the relationships between vessels and bronchi with the lung parenchyma in its natural inflated state. In contrast, it is sometimes difficult to analyze such anatomical relationships by conventional anatomical techniques using resected lung specimens usually in the deflated state.

We also identified some uncommon drainage patterns of veins, such as aberrant V^4+5^, aberrant V^4^ or V^5^, and aberrant V^6^. In particular, aberrant V^6^ was not identified in previous reports. These minor anatomical variations in pulmonary veins can cause serious problems in patients undergoing lung surgery [7, 8]. For example, during right lower lobectomy in a patient with aberrant V^4+5^, if we cut an IPV with an aberrant V^4+5^, the right middle lobe would become dysfunctional after surgery. Furthermore, during subcarinal lymph node dissection in a patient with aberrant V^6^, which runs through the subcarinal area, we may encounter unexpected bleeding if we do not have preoperative knowledge of this uncommon variation. Therefore, knowledge regarding these minor uncommon vessels is necessary to safely perform lung resection.

There were several limitations in this study. First, we could not obtain adequate images of segmental vessels and bronchi from some patients. Although the number of such cases was small, they may have biased our results. Second, our study was an anatomical analysis based on 3DCT findings; therefore, it is possible that there were differences in our data from actual anatomy. The detection rate of vessels by our 3DCTAB was 98.7% [4], so it appeared feasible to compare our 3DCTAB data with those described in previous reports based on actual anatomy. However, the limitation of differences from actual anatomy remains, because we did not compare our data to resected versions of the same lungs.

## Conclusion

This is the first report of variation in the bronchovascular patterns of the RML and RLL with descriptions of rare branching patterns. We extracted data from a large number of 3DCTAB images, which helped elucidate individual variation in thoracic anatomy. Our data were collected with maximum use of 3DCTAB and should represent a valuable reference resource for thoracic surgeons prior to performing lung resection, particularly lobectomy and segmentectomy.

## Electronic supplementary material

Below is the link to the electronic supplementary material.

**Supplemental Fig. 1**. Schema of the right lung from the mediastinal view. S*, an independent segment observed infrequently between S^6^ and S^10^. **Supplemental Fig. 2**. (a)–(c) Types of branching of the arteries in the right middle lobe. (a) Single stem. (b) Two stems. (c) Three stems. (d)–(f) Types of branching of the veins in the right middle lobe. (d) Single stem. (e) Two stems. (f) Three stems. **Supplemental Fig. 3**. Anomalous A^4^ branching pattern; A^4^a branched from A^7^a. **Supplemental Fig. 4**. (a)–(b) Branching patterns of the subsuperior segmental artery (A*) in the right lower lobe. (a) Single stem. (b) Two stems. **Supplemental Fig. 5**. (a)–(e) Branching patterns of the ventrobasal, laterobasal, and dorsobasal segmental arteries (A^8^, A^9^, A^10^) in the right lower lobe. (a) A^8^ and A^9+10^ type. (b) A^8+9^ and A^10^ type. (c) A^8^ and A^8^ + A^9^ + A^10^ type. (d) A^8^ + A^9^ and A^9^ + A^10^ type. (e) A^8^ and A^9^ and A^10^ type. **Figure 6**. (a)–(e) Branching patterns of the ventrobasal, laterobasal, and dorsobasal segmental veins (V^8^, V^9^, V^10^) in the right lower lobe. (a) V^8^ + V^9^ and V^10^ type. (b) V^8^ and V^9^ + V^10^ type. (c) V^8^ + V^9^ + V^10^ and V^10^ type. (d) V^8^ + V^9^ and V^9^ + V^10^ type. (e) V^8^ and V^9^ and V^10^ type. **Figure 7**. (a)–(b) Branching patterns of the superior segmental bronchus (B^6^) in the right lower lobe. (a) Single stem. (b) Two stems: (c)–(e) Branching patterns of the ventrobasal, laterobasal, and dorsobasal segmental bronchi (B^8^, B^9^, B^10^) in the right lower lobe. (c) B^8^ and B^9^ + B^10^ type. (d) B^8^ + B^9^ and B^10^ type. (e) B^8^ and B^9^ and B^10^ type. (PPTX 9712 KB)

Supplementary material 2 (DOCX 37 KB)

## Abbreviations

IPV: Inferior pulmonary vein
MDCT: Multidetector row computed tomography
RLL: Right lower lobe
RML: Right middle lobe
SPV: Superior pulmonary vein
3DCTAB: Three-dimensional CT angiography and bronchography
