# Supplementary material for: Analysis of variation in bronchovascular pattern of the right middle and lower lobes of the lung using three-dimensional CT angiography and bronchography
Source: Gen Thorac Cardiovasc Surg. 2017 Feb 14;65(6):343–9. doi: 10.1007/s11748-017-0754-4 (PMC5437148; doi:10.1007/s11748-017-0754-4)
Supplement: Supplementary file 1 — Supplemental Fig. 1. Schema of the right lung from the mediastinal view. S*, an independent segment observed infrequently between S6 and S10. Supplemental Fig. 2. (a)–(c) Types of branching of the arteries in the right middle lobe. (a) Single stem. (b) Two stems. (c) Three stems. (d)–(f) Types of branching of the veins in the right middle lobe. (d) Single stem. (e) Two stems. (f) Three stems. Supplemental Fig. 3. Anomalous A4 branching pattern; A4a branched from A7a. Supplemental Fig. 4. (a)–(b) Branching patterns of the subsuperior segmental artery (A*) in the right lower lobe. (a) Single stem. (b) Two stems. Supplemental Fig. 5. (a)–(e) Branching patterns of the ventrobasal, laterobasal, and dorsobasal segmental arteries (A8, A9, A10) in the right lower lobe. (a) A8 and A9+10 type. (b) A8+9 and A10 type. (c) A8 and A8 + A9 + A10 type. (d) A8 + A9 and A9 + A10 type. (e) A8 and A9 and A10 type. Figure 6. (a)–(e) Branching patterns of the ventrobasal, laterobasal, and dorsobasal segmental veins (V8, V9, V10) in the right lower lobe. (a) V8 + V9 and V10 type. (b) V8 and V9 + V10 type. (c) V8 + V9 + V10 and V10 type. (d) V8 + V9 and V9 + V10 type. (e) V8 and V9 and V10 type. Figure 7. (a)–(b) Branching patterns of the superior segmental bronchus (B6) in the right lower lobe. (a) Single stem. (b) Two stems: (c)–(e) Branching patterns of the ventrobasal, laterobasal, and dorsobasal segmental bronchi (B8, B9, B10) in the right lower lobe. (c) B8 and B9 + B10 type. (d) B8 + B9 and B10 type. (e) B8 and B9 and B10 type. (PPTX 9712 KB) [file 11748_2017_754_MOESM1_ESM.pptx]

## Slide 1
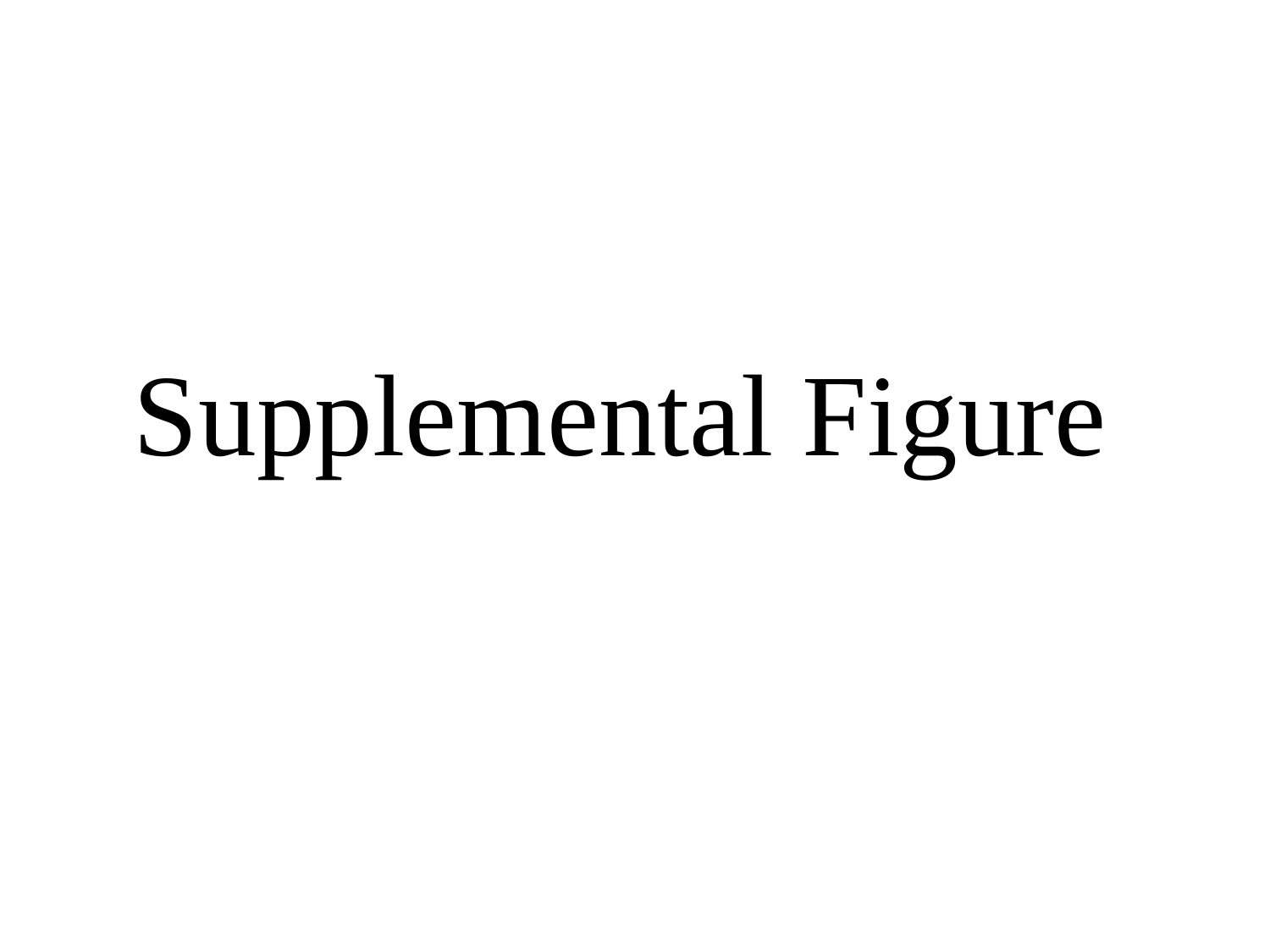

# Supplemental Figure

## Slide 2
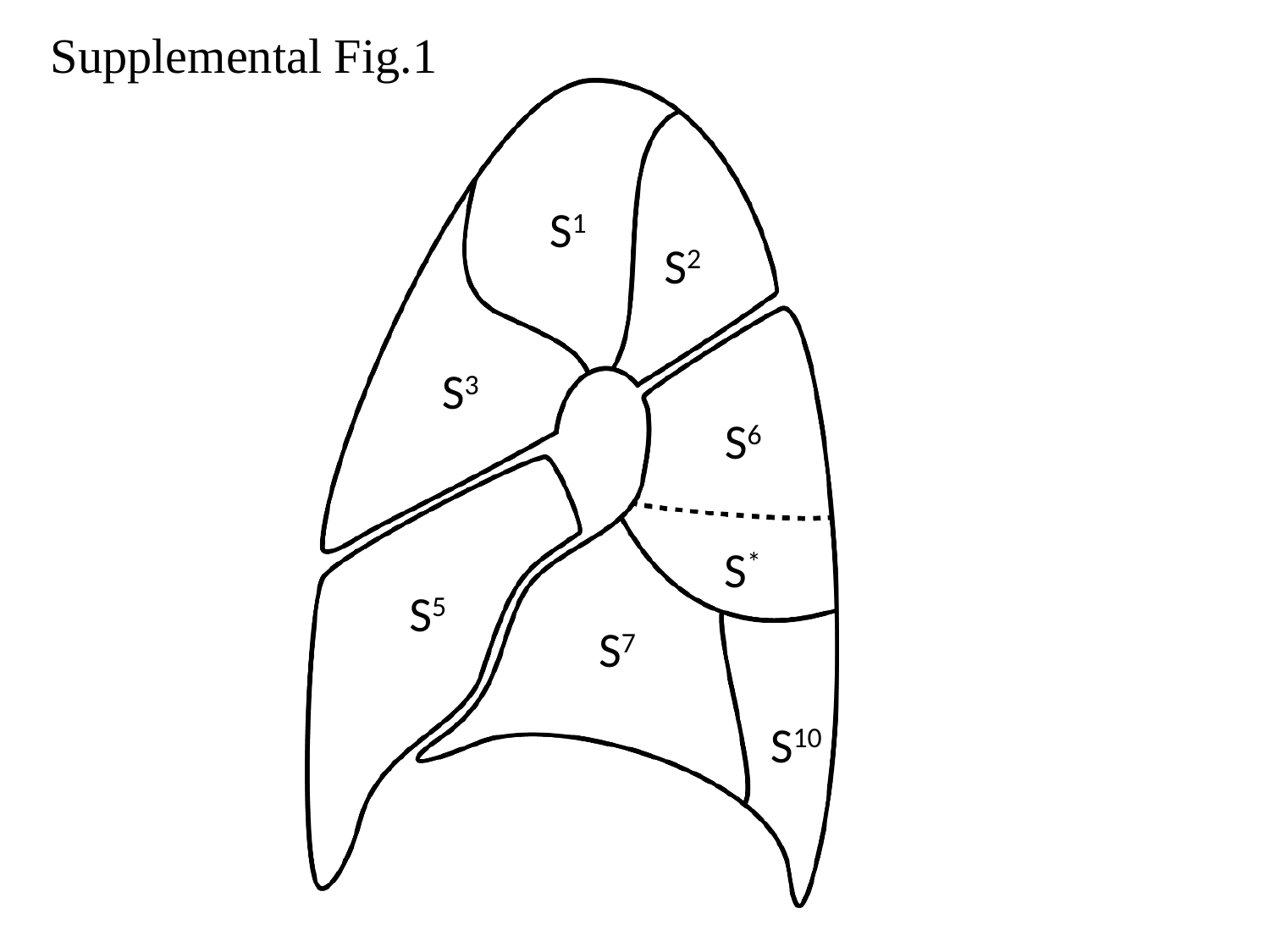

# Supplemental Fig.1
S1
S2
S3
S6
S*
S5
S7
S10

## Slide 3
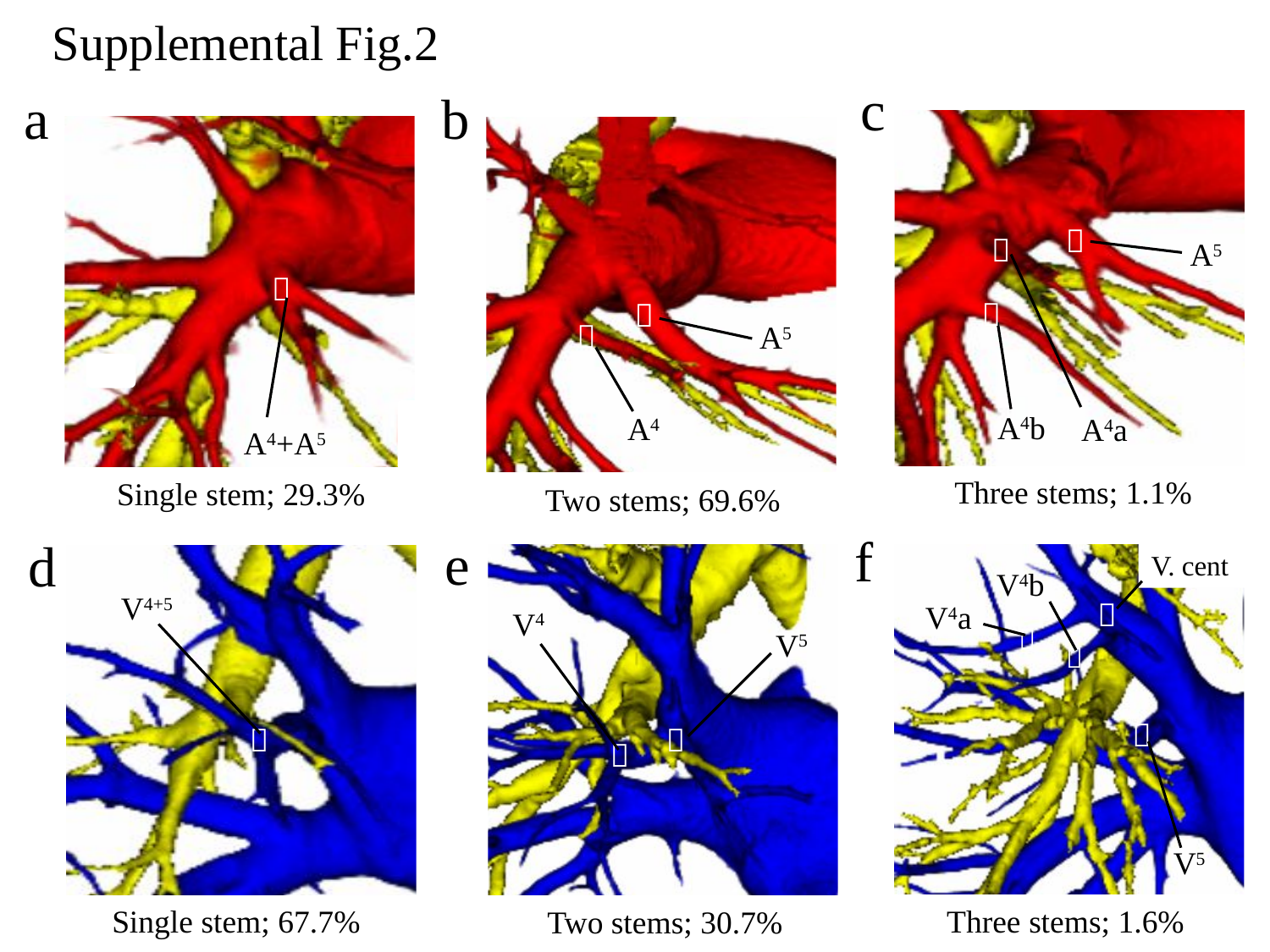

# Supplemental Fig.2
c
a
b
＊
＊
A5
A4b
A4a
＊
Three stems; 1.1%
＊
A4+A5
Single stem; 29.3%
＊
＊
A5
A4
Two stems; 69.6%
f
e
d
V. cent
V4+5
＊
Single stem; 67.7%
V4b
＊
V4a
V4
＊
V5
＊
＊
＊
＊
V5
Three stems; 1.6%
Two stems; 30.7%

## Slide 4
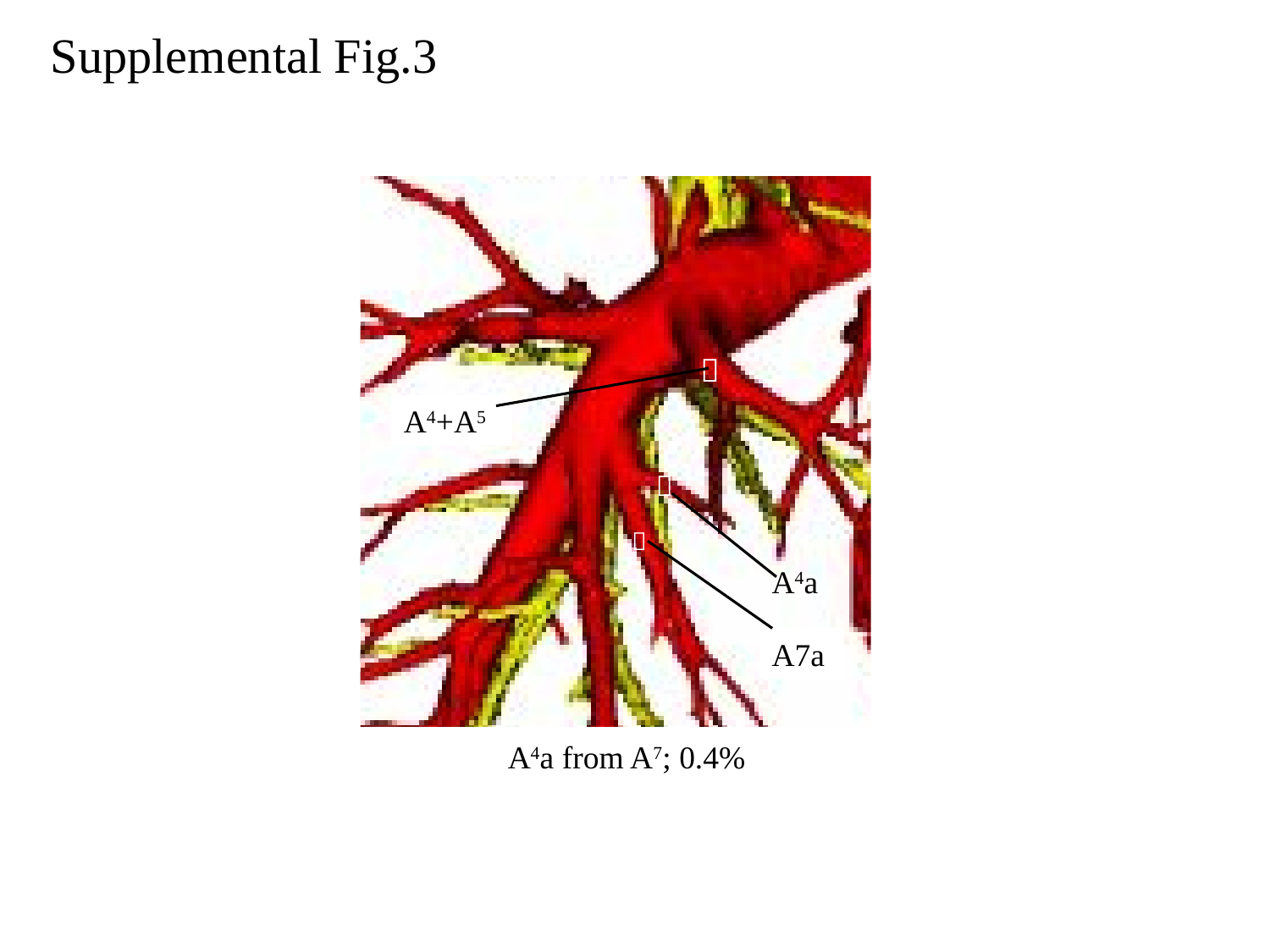

# Supplemental Fig.3
＊
A4+A5
＊
＊
A4a
A7a
A4a from A7; 0.4%

## Slide 5
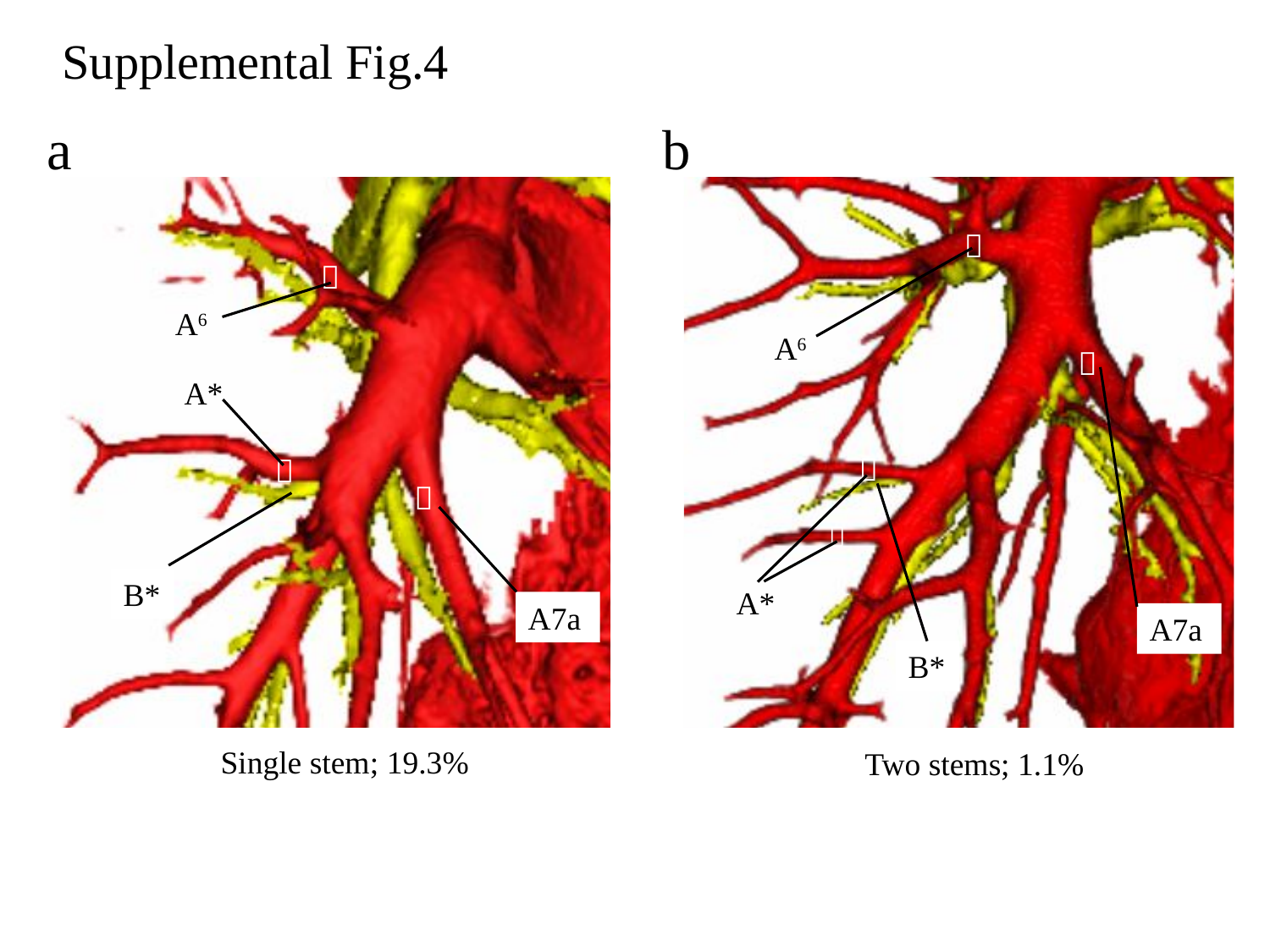

# Supplemental Fig.4
a
b
＊
＊
A6
A6
＊
A*
＊
＊
＊
＊
B*
A*
A7a
A7a
B*
Single stem; 19.3%
Two stems; 1.1%

## Slide 6
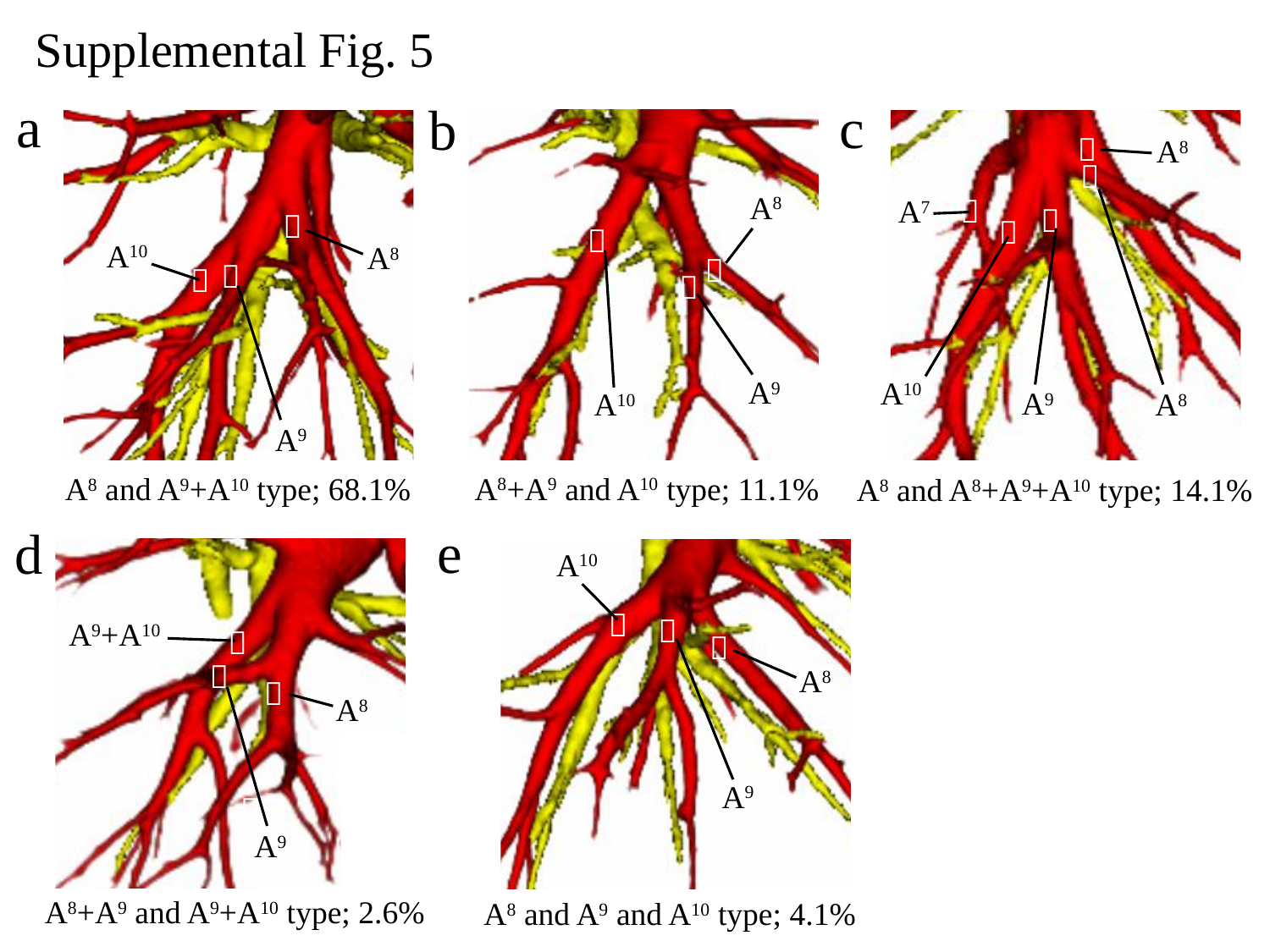

# Supplemental Fig. 5
a
c
b
A8
＊
＊
＊
A9
A10
A8+A9 and A10 type; 11.1%
＊
A8
＊
＊
＊
A10
A9
A8
＊
A7
A8 and A8+A9+A10 type; 14.1%
＊
A10
A8
＊
＊
A9
A8 and A9+A10 type; 68.1%
e
d
A10
＊
＊
＊
A8
A9
A8 and A9 and A10 type; 4.1%
A9+A10
＊
＊
＊
A8
＊
A9
A8+A9 and A9+A10 type; 2.6%

## Slide 7
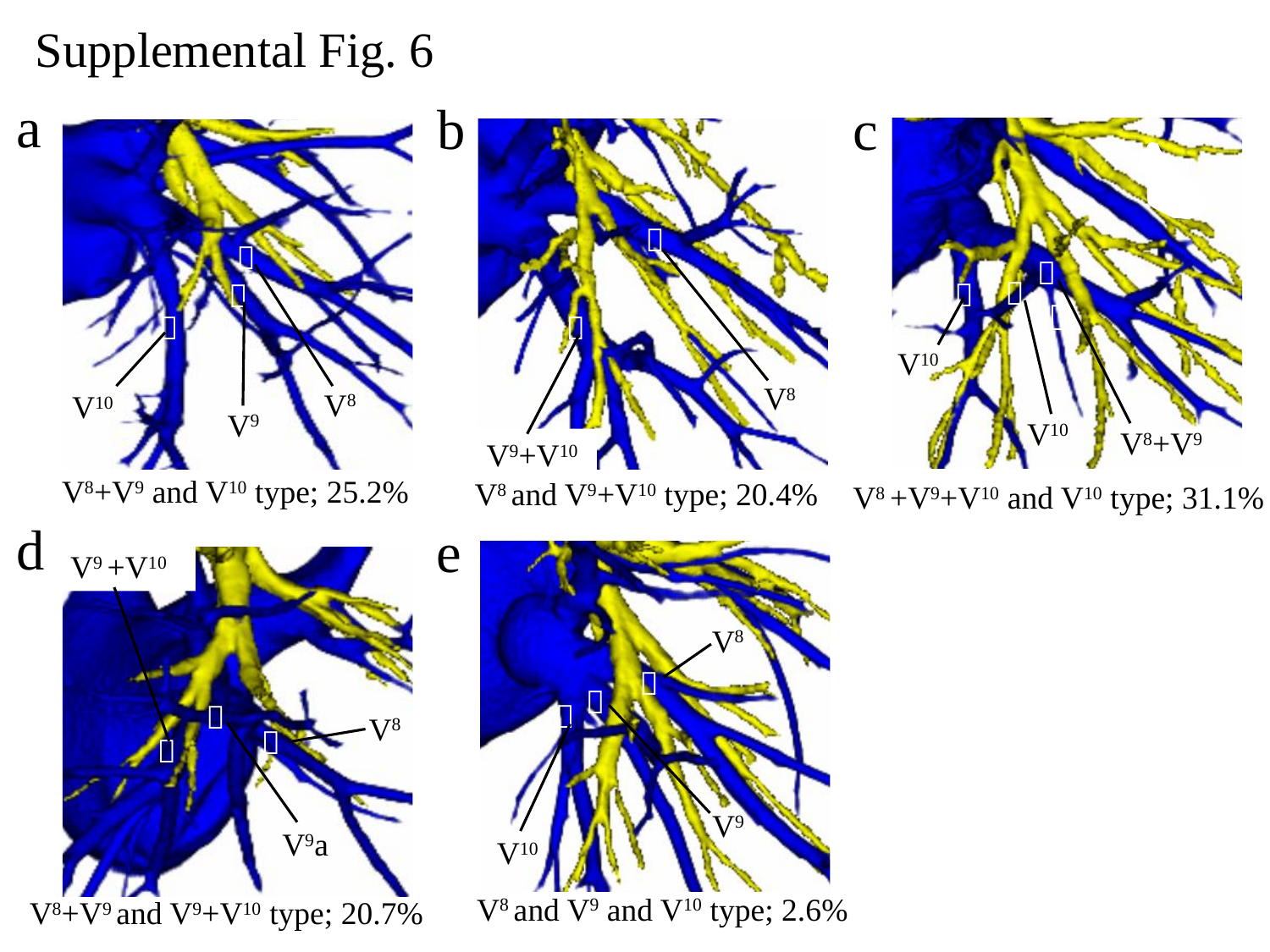

Supplemental Fig. 6
a
b
c
＊
＊
＊
V8
V10
V9
＊
＊
＊
＊
V10
V10
V8+V9
＊
＊
V8
V9+V10
V8+V9 and V10 type; 25.2%
V8 and V9+V10 type; 20.4%
V8 +V9+V10 and V10 type; 31.1%
d
e
V9 +V10
V8
＊
＊
＊
V9
V10
＊
V8
＊
＊
V9a
V8 and V9 and V10 type; 2.6%
V8+V9 and V9+V10 type; 20.7%

## Slide 8
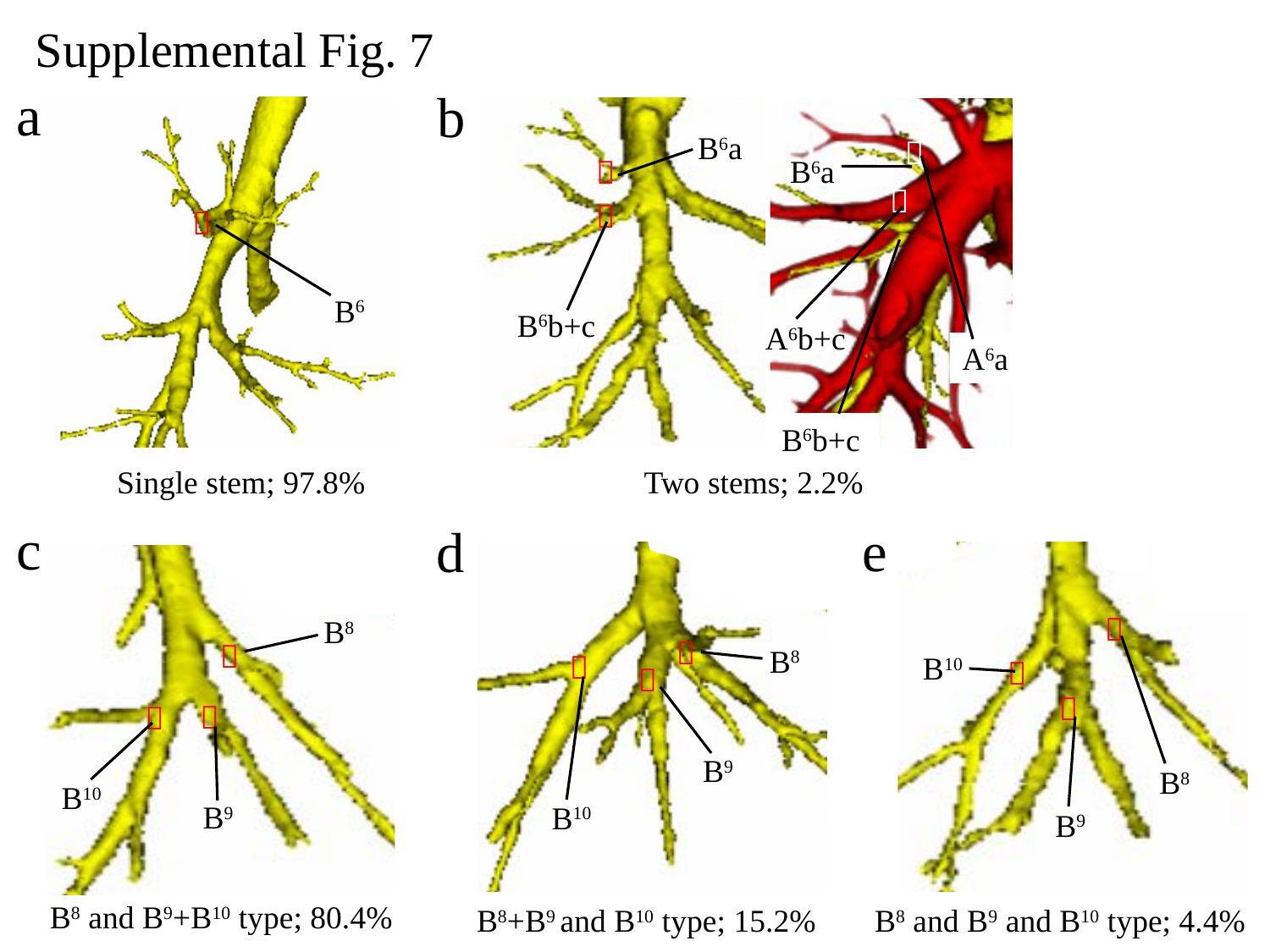

Supplemental Fig. 7
a
b
B6a
＊
B6b+c
＊
B6a
＊
＊
＊
B6
A6b+c
A6a
B6b+c
Two stems; 2.2%
Single stem; 97.8%
c
e
d
＊
B8
＊
＊
B9
B10
＊
B10
＊
＊
B8
B9
B8
＊
＊
＊
B10
B9
B8 and B9+B10 type; 80.4%
B8 and B9 and B10 type; 4.4%
B8+B9 and B10 type; 15.2%
