# Supplementary material for: Analysis of variation in bronchovascular pattern of the right middle and lower lobes of the lung using three-dimensional CT angiography and bronchography
Source: Gen Thorac Cardiovasc Surg. 2017 Feb 14;65(6):343–9. doi: 10.1007/s11748-017-0754-4 (PMC5437148; doi:10.1007/s11748-017-0754-4)
Supplement: Supplementary file 2 — Supplementary material 2 (DOCX 37 KB) [file 11748_2017_754_MOESM2_ESM.docx]

Supplemental Table 1. Summary of patient characteristics.

|  | Cases (*n* = 270) | |
| --- | --- | --- |
|  | No. | % |
| Age (year) |  |  |
| Median (range) | 67 (18-91) |  |
| Gender |  |  |
| Male | 150 | 56 |
| Female | 120 | 44 |
| Disease |  |  |
| Primary lung cancer | 185 | 69 |
| Mediastinal tumor | 28 | 10 |
| Metastatic lung cancer | 28 | 10 |
| Others | 29 | 11 |

Supplemental Table 2. Branching patterns of the right middle lobe vessels and bronchus.

| Middle lobe artery | Our study (*n* = 270) | | Yamashita (*n* = 120) | | *p*-value | Boyden (*n* = 50) | | *p*-value | Fig. |
| --- | --- | --- | --- | --- | --- | --- | --- | --- | --- |
|  | No. | % | No. | % |  | No. | % |  |  |
| Single stem | 79 | 29.3 | 51 | 42.5 | 0.01 | 24 | 48 | 0.009 | s-2a |
| Two stems | 188 | 69.6 | 64 | 53.4 | 0.002 | 26 | 52 | 0.015 | s-2b |
| Three stems | 3 | 1.1 | 5 | 4.1 | 0.049 | NR | - | - | s-2c |
| Middle lobe vein |  | |  | |  |  | |  |  |
| Single stem | 177 | 67.7 | 55 | 45.6 | 0.002 | 26 | 52 | 0.068 | s-2d |
| Two stems | 87 | 30.7 | 48 | 40.0 | 0.136 | 18 | 36 | 0.601 | s-2e |
| Three stems | 6 | 1.6 | 10 | 8.8 | 0.005 | 6 | 12 | 0.001 | s-2f |
| V^4+5^ drain into IPV | 11 | 4.1 | 6 | 4.8 | 0.679 | 4 | 8 | 0.809 | 1a |
| V^4^ or V^5^ drain into IPV | 8 | 3.0 | 1 | 0.8 | 0.196 |  |  |  | 1b |
| Middle lobe bronchus |  | |  | |  |  | |  |  |
| Single stem | 270 | 100 | 120 | 100 | - | 50 | 100 | - | - |

NR ­­= not reported; IPV = inferior pulmonary vein

Supplemental Table 3. Branching patterns of the segmental arteries in the right lower lobe.

| **A^6^** | **Our study (*n* = 270)** | | **Yamashita (*n* = 130)** | | ***p*-value** | **Boyden (*n* = 50)** | | ***p*-value** | **Fig.** |
| --- | --- | --- | --- | --- | --- | --- | --- | --- | --- |
|  | No. | % | No. | % |  | No. | % |  |  |
| Single stem | 223 | 82.6 | 103 | 79.2 | 0.417 | 40 | 80 | 0.660 | 2a |
| Two stems | 46 | 17.0 | 26 | 20.0 | 0.470 | 8 | 16 | 0.857 | 2b |
| Three stems | 1 | 0.4 | 1 | 0.8 | 0.596 | 2 | 4 | 0.014 | 2c |
| **A*** |  | |  | |  |  | |  |  |
| Single stem | 52 | 19.3 | 30 | 23.4 | 0.376 | 24 | 48 | < 0.001 | s-4a |
| Two stems | 3 | 1.1 | 6 | 4.6 | 0.027 | NR | - | - | s-4b |
| A* absence | 215 | 79.6 | 94 | 72.0 | 0.102 | 26 | 52 | < 0.001 | - |
| **A^7^** |  | |  | |  |  | |  |  |
| A^7^a type | 202 | 74.8 | 70 | 53.7 | < 0.001 | 28 | 56 | 0.007 | 3a |
| A^7^ab type | 40 | 14.8 | 37 | 28.5 | 0.001 | 12 | 24 | 0.106 | 3b |
| A^7^b type | 13 | 4.8 | 9 | 6.9 | 0.386 | 7 | 14 | 0.014 | 3c |
| AX^7^ type | 15 | 5.6 | 4 | 3.1 | 0.275 | 3 | 6 | 0.900 | 3d |
| **A^8-10^** |  | |  | |  |  | |  |  |
| Bifurcation | 259 | 95.9 | 127 | 97.7 | 0.368 | 38 | 76 | < 0.001 |  |
| Simple bifurcation type | 214 | 79.2 | 127 | 97.7 | < 0.001 | 38 | 76 | 0.605 |  |
| A^8^ and A^9^+A^10^ | 184 | 68.1 | 118 | 90.8 | < 0.001 | 32 | 64 | 0.565 | s-5a |
| A^8^+A^9^ and A^10^ | 30 | 11.1 | 9 | 6.9 | 0.186 | 6 | 12 | 0.855 | s-5b |
| Split bifurcation type | 45 | 16.7 | NR | - | - | NR | - | - |  |
| A^8^ and A^8^+A^9+^A^10^ | 38 | 14.1 | NR | - | - | NR | - | - | s-5c |
| A^8^+A^9^ and A^9^+A^10^ | 7 | 2.6 | NR | - | - | NR | - | - | s-5d |
| Trifurcation (A^8^, A^9^, A^10^) | 11 | 4.1 | 3 | 2.3 | 0.368 | NR | - | - | s-5e |

NR ­­= not reported

Supplemental Table 4. Branching types of the right lower lobe veins.

| V^6^ | Our study (*n* = 270) | | Yamashita (*n* = 130) | | *p*-value | Boyden (*n* = 50) | | *p*-value | Fig. |
| --- | --- | --- | --- | --- | --- | --- | --- | --- | --- |
|  | No. | % | No. | % |  | No. | % |  |  |
| Single stem | 234 | 86.7 | 110 | 84.5 | 0.580 | 48 | 96 | 0.061 | 2d |
| Two stems | 36 | 13.3 | 20 | 15.5 |  | 2 | 4 |  | 2e |
| V^6^ drain into SPV | 4 | 1.5 | NR | - | - | NR | - | - | 2f |
| V^8-10^ |  | |  | |  |  | |  |  |
| Bifurcation | 263 | 97.4 | 122 | 93.5 | 0.079 | 50 | 100 | 0.250 |  |
| Simple bifurcation type | 123 | 45.6 | 51 | 39.0 | 0.291 | 19 | 38 | 0.372 |  |
| V^8^+V^9^ and V^10^ | 68 | 25.2 | 36 | 27.6 | 0.592 | 16 | 32 | 0.314 | s-6a |
| V^8^ and V^9^+V^10^ | 55 | 20.4 | 15 | 11.4 | 0.044 | 3 | 6 | 0.020 | s-6b |
| Split bifurcation type | 140 | 51.8 | 71 | 54.5 | 0.704 | 31 | 62 | 0.220 |  |
| V^8^+V^9^+V^10^ and V^10^ | 84 | 31.1 | 19 | 14.2 | < 0.001 | 7 | 14 | 0.014 | s-6c |
| V^8^+V^9^ and V^9^+V^10^ | 56 | 20.7 | 44 | 33.8 | 0.005 | 22 | 44 | < 0.001 | s-6d |
| V^8^ and V^8^+V^9^+V^10^ | 0 | 0 | 8 | 6.5 | - | 2 | 4 | - | - |
| Trifurcation (V^8^, V^9^, V^10^) | 7 | 2.6 | 8 | 6.5 | 0.079 | NR | - | - | s-6e |

NR ­­= not reported

Supplemental Table 5. Branching patterns of the segmental bronchi in the right lower lobe.

| **B^6^** | **Our study (*n* = 270)** | | **Yamashita (*n* = 180)** | | ***p*-value** | **Boyden (*n* = 50)** | | ***p*-value** | **Fig.** |
| --- | --- | --- | --- | --- | --- | --- | --- | --- | --- |
|  | No. | % | No. | % |  | No. | % |  |  |
| Single stem | 264 | 97.8 | 180 | 100 | 0.044 | 46 | 92 | 0.031 | s-7a |
| Two stems | 6 | 2.2 | 0 | 0 |  | 4 | 8 |  | s-7b |
| **B*** |  | |  | |  |  | |  |  |
| Single stem | 55 | 20.4 | 41 | 22.7 | 0.541 | 24 | 48 | < 0.001 | s-4a |
| Two stem | 0 | 0 | 10 | 5.4 | < 0.001 | NR | - | - | - |
| B* absent | 215 | 79.6 | 129 | 71.9 | 0.051 | 26 | 52 | < 0.001 | - |
| **B^7^** | **Our study (*n* = 270)** | | **Yamashita (*n* = 130)** | |  | **Boyden (*n* = 50)** | |  |  |
| B7a type | 202 | 74.8 | 70 | 53.7 | < 0.001 | 11 | 22 | < 0.001 | 3a |
| B7ab type | 40 | 14.8 | 37 | 28.5 | 0.001 | 29 | 58 | < 0.001 | 3b |
| B7b type | 13 | 4.8 | 13 | 10.0 | 0.049 | NR | - | - | 3c |
| BX7 type | 15 | 5.6 | 10 | 7.8 | 0.408 | 10 | 20 | < 0.001 | 3d |
| **B^8-10^** |  | |  | |  |  | |  |  |
| B^8^ and B^9^+B^10^ | 217 | 80.4 | 118 | 90.8 | 0.008 | 47 | 94 | 0.020 | s-7c |
| B^8^+B^9^ and B^10^ | 41 | 15.2 | 9 | 6.9 | 0.019 | NR | - | - | s-7d |
| B^8^ and B^9^ and B^10^ | 12 | 4.4 | 3 | 2.3 | 0.292 | NR | - | - | s-7e |

NR ­­= not reported
